# Transvaginal morcellation within an enclosed bag in gynecological surgeries: a comprehensive systematic review and analysis of safety, efficacy, and outcomes

**DOI:** 10.1007/s00404-024-07676-y

**Published:** 2024-08-03

**Authors:** Omar Alomari, Muhammed Edib Mokresh, Emir Muvaffak, Rahime Nurbanu Bakir, Raghad Al Shomali, Serkan Akis, Sami Acar, Murat Api

**Affiliations:** 1 Hamidiye International Faculty of Medicine, University of Health Sciences, Istanbul, 34668 Türkiye; 2 Faculty of Medicine, Department of Gynecologic Oncology, Marmara University, Pendik Training and Research Hospital, Istanbul, Türkiye; 3 Faculty of Medicine, Department of General Surgery, Tekirdağ Namık Kemal University, 59100 Terkirdağ, Türkiye; 4 Department of Gynecologic Oncology, University of Health Sciences Turkey, Kartal Dr. Lutfi Kirdar Training and Research Hospital, Istanbul, Türkiye

**Keywords:** Safety, Transvaginal, In-bag, Surgical complications, Gynecological surgery

## Abstract

**Purpose:**

This systematic review aims to comprehensively assess the safety and efficacy of transvaginal morcellation within an enclosed bag in gynecological surgeries, with a focus on its benefits, potential risks, and recommendations for its use.

**Methods:**

We conducted a comprehensive search of Epistemonikos, Web of Science, Medline (PubMed), Scopus, and Cochrane databases for studies on transvaginal contained morcellation in adult patients undergoing gynecological surgeries. The review included 22 studies that met the inclusion criteria, encompassing diverse surgical procedures, patient profiles, and outcomes. These studies were thoroughly reviewed and analyzed to assess the safety and efficacy of the morcellation technique.

**Results:**

Key findings from the selected studies indicate that transvaginal morcellation within an enclosed bag offers several advantages in gynecological surgeries, including reduced invasiveness, shorter operative times, and minimal blood loss when compared to conventional methods. The risk of tumor recurrence or dissemination appears to be low when appropriate precautions are taken, emphasizing the technique’s safety, especially when performed by experienced surgical teams. While some studies reported complications, these were generally not directly associated with the morcellation technique.

**Conclusion:**

Transvaginal morcellation within an enclosed bag demonstrates potential as a safe and effective option for gynecological surgeries. The technique offers the benefits of minimally invasive procedures, including reduced bleeding, shorter recovery times, and improved cosmetic outcomes. This review also highlights the need for standardization in study methodologies and reporting, as the heterogeneity in outcomes across the selected studies poses challenges in drawing definitive conclusions.

## Introduction

Morcellation, a minimally invasive surgical technique used in gynecology, involves the removal of tissue or masses from the pelvic area. This approach has gained prominence due to its ability to reduce bleeding, minimize post-surgery pain, shorten recovery periods, and provide improved cosmetic results compared to traditional open surgery [1]. However, concerns arose with the adoption of uncontained power morcellation, which employs rapidly rotating blades to cut tissue into smaller pieces for removal through a port [2]. This technique posed risks of disseminating tissue fragments within the abdominal cavity, especially when dealing with undiagnosed sarcomas [3]. As a response to these concerns, the U.S. Food and Drug Administration (FDA) issued a safety warning in 2014, urging healthcare providers to discuss alternative treatment approaches with patients [4].

In light of the FDA’s warning, gynecological surgeons have explored safer alternatives such as contained transvaginal morcellation [5]. This method aims to maintain the benefits of minimally invasive surgery while reducing the risk of tissue dissemination. Previous studies have shown that there were few complications and good postoperative outcomes, even though there has been ongoing discussion about the duration of morcellation in these procedures [6, 7]. Since a significant portion of patients needed morcellation to complete their surgeries, transvaginal morcellation spared many patients from the potential complications associated with laparotomy [7]. These advancements highlight the convergence of innovation and patient well-being, offering women safer and more efficient surgical choices.

Several critical knowledge gaps persist in the understanding of transvaginal morcellation within an enclosed bag in gynecological surgeries. These gaps encompass various aspects, such as the optimal patient selection criteria, the potential impact of tissue characteristics on the procedure, and the long-term outcomes in terms of tumor recurrence and patient quality of life. Furthermore, the heterogeneity in study methodologies and patient populations has contributed to variations in reported outcomes, making it challenging to establish standardized protocols and guidelines for this technique. Filling these knowledge gaps is of paramount importance as it can lead to improved patient safety, more informed decision-making by clinicians, and the development of evidence-based practices in gynecological surgery. Addressing these gaps can also help mitigate potential risks associated with the procedure and enhance its overall effectiveness.

The primary aim of this study is to conduct a systematic and comprehensive review of the existing literature on transvaginal morcellation within an enclosed bag in gynecological surgeries. By synthesizing the available evidence, we seek to provide a thorough evaluation of the technique’s safety and efficacy across various surgical scenarios and patient populations. This review will critically analyze primary and secondary outcomes, including procedure duration, blood loss, reported adverse events, and long-term follow-up data. Through this investigation, we aim to identify areas of consensus and divergence in the current literature, offer insights into the factors influencing surgical outcomes, and highlight any potential concerns or areas for improvement. Ultimately, our study aims to contribute to the development of evidence-based guidelines for the use of transvaginal morcellation within an enclosed bag in gynecological surgeries, promoting safer and more effective minimally invasive approaches in the field.

## Methods

We used a systematic review strategy to perform complete literature retrieval across several academic databases to explore scientific papers relevant to the outcomes of contained transvaginal morcellation. This review followed the Preferred Reporting Items for Systematic Reviews and Meta-Analysis (PRISMA) 2020 standards, guaranteeing a robust and uniform methodology [8]. We submitted the research protocol for this systematic review to the International Prospective Registry of Systematic Reviews (PROSPERO) database (www.crd.york.ac.uk/prospero/) and assigned the PROSPERO ID: CRD42023437418. All working group members agreed on the study protocol before beginning the literature search.

### Search strategy

An electronic search was conducted across five databases: Epistemonikos, Web of Science, Medline (through PubMed), Scopus, and Cochrane. In July 2023, a search was performed using a strategic combination of relevant terms to ensure effective results. The search strategy included the following terms: (morcellation OR morcellating OR morcellator OR morcellate OR morcellement OR Morcellation* OR Morcellator* OR “Tissue fragmentation”) AND (In-bag OR In-bag OR In-bag* OR within a bag OR enclose OR enclosed OR enclosed bag OR enclosure OR enclosing OR contained OR Intra-corporeal OR Intra-corporeal) AND (transvaginal OR vaginal OR intra-vaginal OR intra-vaginally OR intra-vaginal OR intra-vaginally).

The retrieved studies were carefully assessed based on their titles and abstracts, and only those that met the eligibility criteria were considered for the following stages, which involved extraction and quality assessment. The search for published work was conducted without imposing time restrictions or filters, aiming for inclusivity in the results without limitations based on publication date or predefined criteria. In addition, we conducted a manual search by reviewing the references of the included articles, and literature reviews for possible relevant studies.

### Study selection

The collected articles were checked for potential duplicates. Following the removal of duplicates, the titles and abstracts were screened by four independent reviewers (RA, NB, MEM, and EM). Any discrepancies between the reviewers were resolved by carefully re-examining the article and discussing it until an agreement was achieved. If a resolution could not be reached, a supervisor (MA) was available to re-evaluate the distinctions and make a final decision regarding whether to include the study in the analysis.

**Fig. 1:**
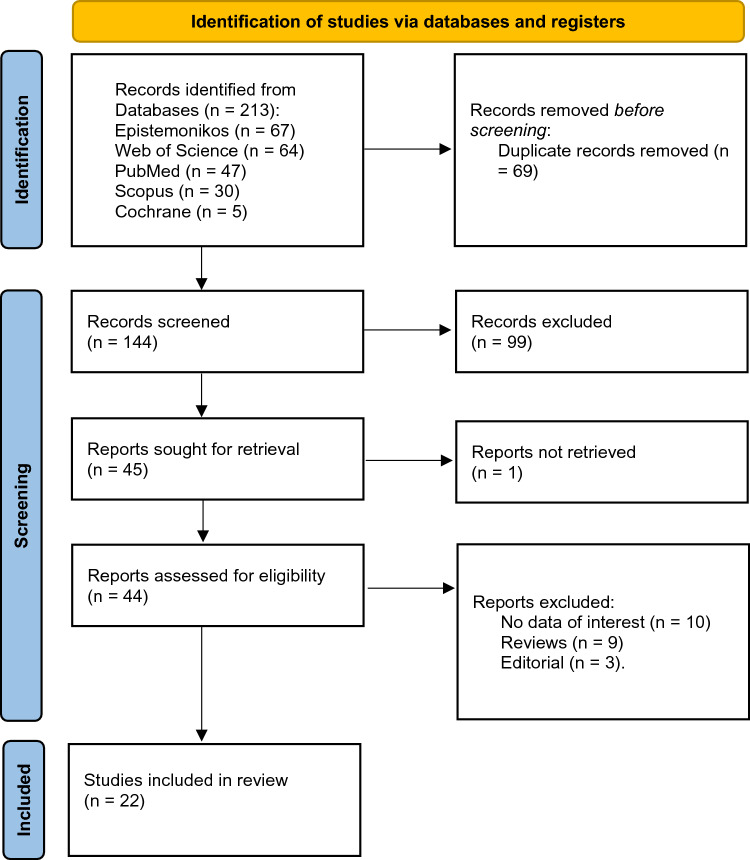
PRISMA 2020 flow diagram for new systematic reviews which included searches of databases and registers only

Studies deemed relevant and any conflicts were subjected to full-text screening. Four reviewers (OA, EM, RA, and NB) obtained the full texts of studies that may have met the criteria and independently assessed them to decide if they should be included in the final analysis. The process for resolving disagreements during the full-text screening was similar to that used previously. The detailed search process and the selection of studies are elaborated in the accompanying PRISMA diagram (Fig. 1).

### Eligibility criteria

This review focused on adult patients who underwent gynecological surgeries involving transvaginal contained morcellation procedures. The selection criteria were not limited by language or study type, aiming to encompass a broad range of research perspectives. The inclusion criteria were all observational studies (prospective or retrospective cohort, case–control, cross-sectional, case series, or case study) and experimental studies (randomized controlled trials).

Our commitment to maintaining the rigor and significance of the assembled studies led us to exclude duplicate publications, reviews, editorial correspondence, book chapters, animal and laboratory experiments, and in vitro inquiries.

### Data extraction

Four authors, RA, EM, NB, and MEM, conducted primary data extraction, each assigned to a specific portion of the studies. They entered the collected information into a pre-piloted Excel spreadsheet. To ensure data accuracy and consistency, a fifth (OA) author meticulously reviewed the completed extraction sheet, reconciled any discrepancies, and validated the precision of the data. The extracted information included details about the authors, publication years, countries of publication, study types, sample sizes, bag type, surgery indications, the type of surgical procedures, duration of the procedure, amount of blood loss, reported adverse events and the main findings of the included studies.

### Critical appraisal tool and risk of *bias* assessment

Two authors (MEM and OA) conducted an assessment of the risk of bias in the eligible included studies. The National Institutes of Health (NIH) Quality Assessment Tool for Observational Cohort and Cross-sectional Studies was employed for this purpose [9]. The assessment involved rating studies on a scale of 0 for poor (0–4 out of 14 questions), for fair (5–10 out of 14 questions), and good (11–14 out of 14 questions). In cases where certain questions were not applicable (NA) or not reported (NR), these designations were used accordingly. The methodological quality of the included clinical trial was assessed by using the RoB2 tool [10]. Any discrepancies in their assessments were resolved through discussions among researchers until a consensus was reached.

## Results

### Summary of included studies

**Fig. 2:**
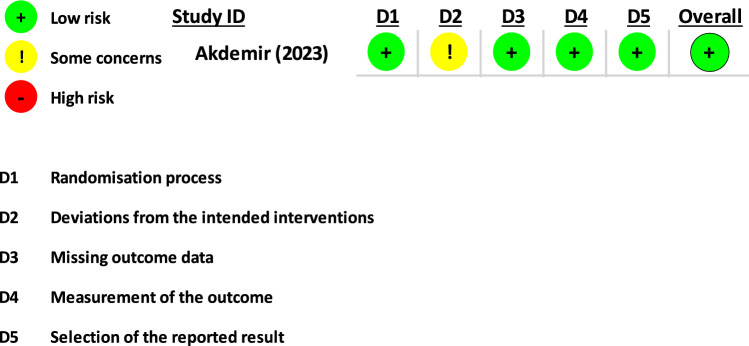
The results of the RoB2 (Risk of Bias 2) Cochrane tool assessment for the included randomized trial conducted across five key domains

The electronic search yielded results from five different databases: Epistemonikos (67 hits), Web of Science (64 hits), MEDLINE through PubMed (47 hits), Scopus (30 hits), and Cochrane (five hits). After inclusion, exclusion, and deletion of duplicates, 22 articles were included in the final analysis [11–32]. The present study identified several published studies including clinical trials, retrospective studies, and case series. Table 1 summarizes the findings of these studies. The present study identified studies related to the safety and effectiveness of transvaginal morcellation within an enclosed bag in gynecological surgeries as well as the risk of tumor recurrence or dissemination. The overall quality of the studies ranged from fair to good, although one study exhibited poor methodology and was designated as having a high risk of bias. A comprehensive breakdown of the quality assessment results is presented in Table 2 and Fig. 2.

**Table 1:** Summary and baseline characteristics of the included studies

Author (year)	Country	Study type	Sample size	Histologic type/indications	Operation	Bag type	Summary of effects or relevant findings	Adverse events
Akdemir et al. [11]	Turkey	*Randomized clinical trials*	48	Abnormal uterine bleeding 21 (43.7%), pelvic pain 12 (25%), infertility 11 (23%), dyspareunia 1 (2.1%), and dysmenorrhea 3 (6.2%)	Laparoscopic myomectomy	Endoscopic specimen bag	The pivotal aspect of Vaginal-enclosed scalpel morcellation was the careful placement of myoma into an endoscopic bag and its retrieval through the posterior colpotomy, ensuring the containment and controlled extraction of tissue. This technique not only impacts surgical efficiency but also has important implications for patient outcomes, emphasizing the importance of employing such containment measures in minimally invasive gynecological surgery	None
Carrubba et al. [12]	USA	Cohort	29	Leiomyomas	Laparoscopic myomectomy	Alexis Contained Extraction System, Applied Medical, Rancho Santa Margarita, CA, USA	Larger uterine dimensions were found to be correlated with longer total operating and morcellation times. However, uterine size and volume, as determined by preoperative imaging, did not exhibit a significant association with the choice of morcellation route, although a trend in this direction was observed	Postoperative complications included readmission for pulmonary emboli in one patient and surgical repair for vaginal cuff separation in another, with benign findings at final pathology
Casarin et al. [13]	Italy	Case series	10	5 were Uterine fibroids	Laparoscopic subtotal hysterectomy	Endo Catch II	Laparoscopic supracervical hysterectomy followed by in-bag transvaginal specimen extraction demonstrates promise as a technique and could be regarded as a reliable and safe option to further minimize the invasiveness of the procedure	One patient experienced postoperative vaginal bleeding and sought emergency care, but no pathologic findings were identified
Chandler et al. [14]	USA	Case series	16	4 Adenomyosis	Laparoscopic hysterectomy	NA	Contained vaginal extraction provides a viable alternative to total abdominal hysterectomy and should be utilized whenever feasible	None
Cohen et al. [15]	USA	Cohort	32	Benign in all patients	Laparoscopic hysterectomy	Ecosac, Lahey Bag, Endo Catch, Anchor tissue retrieval system, Alexis Contained Extraction System	In terms of tissue extraction method, both contained minilaparotomy and contained vaginal extraction approaches demonstrate comparable patient outcomes and recovery characteristics	Nine patients experienced postoperative complications, and one patient required readmission
Ding et al. [16]	China	Cohort	1560	Uterine fibroids	Abdominal, vaginal, laparoscopic or robot-assisted hysterectomies	NA	Abdominal hysterectomy with in-bag morcellation proves to be a time-efficient and feasible alternative to laparotomy, serving as a valuable option until more advanced alternatives are developed	None
Dotson et al. [17]	USA	Cohort	81	27 Uterine fibroids	Laparoscopic hysterectomy	Endo Catch specimen retrieval bag	Contained uterine hand morcellation demonstrates feasibility with low peri-operative complication rates, making it a viable option for minimally invasive surgical procedures in cases of large uterine neoplasms. However, further assessment is necessary to evaluate survival outcomes, especially for uterine malignancies	Seven patients experienced postoperative complications, resulting in one readmission. These complications included issues like vaginal cuff separation, port-site hematoma, cellulitis, bladder infection, pulmonary edema, and musculoskeletal injury
English et al. [18]	USA	Case Study	1	Abnormal bleeding	Robotic-assisted total hysterectomy	A bowel bag (3M Steri-DrapeTM Isolation Bag 1003, St. Paul, MN)	Vaginal morcellation of a large uterus was safely performed in a nulliparous patient during a robotic-assisted total hysterectomy, with no intra-abdominal spillage	None
Favero et al. [19]	Brazil	Case series	8	Symptomatic uterine leiomyomas, Pelvic organ prolapse, Symptomatic endometriosis or adenomyosis, other benign conditions (NO SPECİFİC NUMBERS)	Laparoscopic hysterectomy	LapSac	Vaginal morcellation following oncologic principles is a feasible method that facilitates rapid uterine extraction and has the potential to reduce the need for unnecessary laparotomies. However, additional research is required to establish the oncological safety of this technique definitively	There was one reported case of postoperative vesicovaginal fistula
Favero et al. [20]	Brazil	Cohort	30	29 leiomyoma	Laparoscopic hysterectomy	LapSac (Cook Medical, Bloomington, IN)	Although our study had a relatively small sample size and a limited follow-up period, the findings reinforce our preliminary results, indicating that the protected vaginal delivery of a large uterus in women with endometrial cancer could be a viable surgical option with potential oncologicl safety. However, more extensive prospective studies are essential to confirm the validity and safety of this approach comprehensively	Two patients experienced postoperative complications, with one case involving a vesicovaginal fistula and the other presenting with dehiscence of the vaginal cuff suture
Ghezzi et al. [21]	Italy	Cohort	316	52 endometrial cancer	Laparoscopic myomectomy	10- or 15-mm polyurethane specimen pouch (Endo Catch II; Covidien Surgical, Mansfield, MA, USA)	The technique of contained transvaginal extraction of fibroid specimens has demonstrated both safety and efficiency in the majority of women undergoing laparoscopic myomectomy. This approach serves as a valuable and minimally invasive alternative to intracorporeal morcellation, potentially reducing the associated risks and complications	Postoperatively, there were complications observed in 18 cases, accounting for 5.7% of the cases studied
Gil-Gimeno et al. [22]	Canada	Case series	79	Simple hyperplasia without atypia, leiomyomas, adenomyosis and evidence of endometriosis	Total Laparoscopic Hysterectomy	32*41 cm nylon bag (4L Espiner EcoSac morcellation, Espiner Medical) deployed intra-abdominally	In-bag morcellation has shown feasibility in a substantial portion of women undergoing laparoscopic hysterectomy, albeit with a notable increase in operative time. To gain a more comprehensive understanding of the potential complications and benefits, particularly in terms of minimizing tissue dissemination, larger studies will be necessary	Complications were reported in two women who had in-bag morcellation: one case of posterior vaginal laceration during vaginal morcellation and one case of bladder injury in a woman with a uterus of 1321 g that likely occurred during initial bladder dissection
Günthert et al. [23]	Switzerland	Cohort	61	Endometrioid adenocarcinoma- 4 patients	Total Laparoscopic Hysterectomy	Plastic bag (Auto Suture EndoCatch II 15 mm; Covidien/Tyco Healthcare, Norwalk, CT) + For larger uterine specimens, bigger bags without frame (LapSac; Cook Medical, Bloomington, IN) were applied, although the placement and wrapping were more difficult and took much more time	In-bag morcellation techniques should be avoided in cases involving suspected or confirmed malignancy. Although our case series did not report any bag rupture or perforation incidents, it is essential to acknowledge the potential risk associated with morcellation. Performing a thorough histopathology workup and staging for a morcellated uterus in cases of malignancy might present significant challenges	None
Kliethermes et al. [24]	USA	Case Study	1	Serous adenocarcinoma	Total Laparoscopic Hysterectomy	Bag, an Alexis ring, and a stapler	Morcellation performed through the vagina, as demonstrated in this video, offers a quick and straightforward method. This technique not only ensures vaginal protection and retraction but also contains the specimen, preventing its loss during morcellation and minimizing the risk of potential malignancy spread. Furthermore, by eliminating the necessity to close the abdominal fascia, this approach reduces surgical time and alleviates concerns related to hernia formation resulting from extended incisions	None
Meurs et al. [25]	USA	Cohort	24	Carcinosarcoma 1 patient	Full-text not available	Full-text not available	All three morcellation techniques (electronic power morcellation, manual morcellation via the vagina, or manual morcellation via minilaparotomy) are considered viable options for tissue extraction during minimally invasive surgeries, and the choice may depend on individual patient factors and surgical preferences	Postoperatively, adverse effects were observed in 5 patients (9.3%)
Montella et al. [26]	Italy	Case series	12	Clear-cell adenocarcinoma 1 patient	Laparoscopic or robot-assisted laparoscopic hysterectomy or myomectomy	NA	Utilizing vaginal morcellation while adhering to oncologic principles facilitates swift uterine extraction and has the potential to reduce the need for avoidable laparotomies	None
Nakabayashi et al. [27]	Japan	Case Study	1	Endometrioides adenocarcinoma	Total laparoscopic hysterectomy	Sterile plastic wrapping bag	A 55-year-old woman initially diagnosed with uterine leiomyoma underwent laparoscopic hysterectomy with transvaginal morcellation to prevent content spillage, as the MRI showed atypical features resembling a “bag of worms.” Subsequent retrospective MRI analysis revealed a band-like structure. She also had a laparoscopic bilateral salpingo-oophorectomy and remains free of recurrence	None
Serur et al. [28]	USA	Cohort	104	Serous adenocarcinoma 3	Total laparoscopic hysterectomy	LiNA EasyBag	In our five years of experience, manual morcellation within an endoscopic bag has proven to be a safe method for extracting large uteri without the need for a power morcellator. Throughout this period, we have not encountered any instances of visible spillage, bag rupture, or complications related to our morcellation technique.m	One patient had an estimated blood loss of 1200 mL secondary to bleeding from a broad ligament myoma. Eight patients required a postoperative blood transfusion. Of the 3 patients who required a hospitalization of 4 days, 2 had postoperative ileus that resolved with supportive care
Solima et al. [29]	Italy	Cohort	12	Carcinosarcoma 3	Robotic hysterectomy	Endobag	Minor damage to the bag can occur following transvaginal morcellation of uteri that require removal by this method. Such damage has the potential to impact the spread of cancer cells into the abdominal cavity	None
Takeda et al. (2018) [30]	Japan	Case Study	1	Clear-cell carcinoma 1	Laparoscopic supracervical hysterectomy (vaginal)	EndoCatch II	The excised peritoneal mass was enclosed within a retrieval bag and subsequently extracted through the vagina. The pathologic diagnosis confirmed it as a parasitic peritoneal myoma with myxoid degeneration. The postoperative course proceeded without any complications, and there was no recurrence of the parasitic myoma during the one-year follow-up after the surgery	None
Wyman et al. [31]	USA	Case series	100	Abnormal uterine bleeding	Total laparoscopic hysterectomy (abdominal)	(Endo Catch II 15 mm Specimen Pouch, Covidien, Norwalk, CT)	The use of the retrieval bag is particularly noteworthy, as it addresses a critical aspect of specimen retrieval and contributes to the success of complex gynecological procedures	One patient required postoperative secondary surgery
Zhang et al. [32]	China	Cohort	42	Pain and/or pressure	Vaginal morcellation during total laparoscopic hysterectomy	Endo Catch	Morcellation within a disposable extraction bag, aided by a traction wire introduced through the posterior vaginal fornix during laparoscopic myomectomy, emerges as a safe and viable technique for the removal of fibroids. This method holds promise in mitigating the risk of iatrogenic tumor dissemination and offers a potentially valuable approach in gynecological surgery	None

**Table 2:** Quality-assessment tool for observational cohort and cross-sectional studies (NIH)

Refs	Q1	Q2	Q3	Q4	Q5	Q6	Q7	Q8	Q9	Q10	Q11	Q12	Q13	Q14	Overall rating
Carrubba[12]	+	+	+	+	*	+	+	+	+	-	+	*	+	+	Good
Casarin[13]	+	+	+	+	NR	+	+	NA	+	NA	+	NA	*	NA	Good
Cohen[15]	+	+	+	+	+	+	+	+	+	-	+	*	+	*	Good
Ding[16]	+	+	+	+	-	+	+	*	*	NR	+	NR	+	*	Fair
Dotson[17]	+	+	+	+	-	+	+	+	+	NA	+	-	NR	+	Good
Favero[19]	+	+	+	*	-	+	+	NA	+	NA	+	NA	NA	NA	Fair
Favero[20]	+	+	+	+	*	NA	+	+	+	*	+	CD	+	+	Good
Ghezzi[21]	+	+	CD	+	-	+	+	-	+	-	+	NR	*	-	Fair
Gil-Gimeno [22]	+	+	+	+	-	+	+	+	+	NR	+	*	NR	NA	Good
Guenthert[23]	+	+	NR	-	-	-	CD	-	-	NR	NR	-	CD	-	Poor
Meurs[25]	+	+	+	CD	-	+	+	-	+	-	+	-	+	CD	Fair
Montella[26]	*	+	+	+	-	+	CD	NA	+	NA	+	-	NA	-	Fair
Serur[28]	+	+	+	+	-	+	CD	NR	+	-	+	NR	+	-	Good
Solima[29]	+	+	+	+	NR	+	CD	-	+	-	+	NR	NR	CD	Fair
Wyman[31]	+	+	+	+	-	+	+	NA	+	-	+	*	NR	*	Fair
Zhang [32]	+	+	+	+	+	+	+	+	+	NA	+	+	+	+	Good

( +) yes/low risk, (-) no/high risk, (*) unclear, *CD cannot determine, *NA* not applicable, *NR* not reported

Q1: Was the research question or objective in this paper clearly stated?

Q2: Was the study population clearly specified and defined?

Q3: Was the participation rate of eligible persons at least 50%?

Q4: Were all the subjects selected or recruited from the same or similar populations (including the same time period)? Were inclusion and exclusion criteria for being in the study prespecified and applied uniformly to all participants?

Q5: Was a sample size justification, power description, or variance and effect estimates provided?

Q6: For the analyses in this paper, were the exposure(s) of interest measured prior to the outcome(s) being measured?

Q7: Was the timeframe sufficient so that one could reasonably expect to see an association between exposure and outcome if it existed?

Q8: For exposures that can vary in amount or level, did the study examine different levels of the exposure as related to the outcome (e.g., categories of exposure, or exposure measured as continuous variable)?

Q9: Were the exposure measures (independent variables) clearly defined, valid, reliable, and implemented consistently across all study participants?

Q10: Was the exposure(s) assessed more than once over time?

Q11: Were the outcome measures (dependent variables) clearly defined, valid, reliable, and implemented consistently across all study participants?

Q12: Were the outcome assessors blinded to the exposure status of participants?

Q13: Was loss to follow-up after baseline 20% or less?

Q14: Were key potential confounding variables measured and adjusted statistically for their impact on the relationship between exposure(s) and outcome(s)?

### Age and sample size

The age distribution of the participants in the included studies exhibited considerable variability, spanning a median age range of 35–61 years. This wide age range reflects the diverse demographic profiles of the studied patient population. Likewise, the sample sizes across these investigations exhibited substantial heterogeneity, with studies ranging from a single participant to as many as 1560 individuals. This wide variability in sample sizes highlights the diversity in study designs and varying degrees of statistical power across the included studies.

### Bag type and histologic type/indications

Various bag types were utilized in the included studies, with seven studies using “Endo Catch/Endo Catch II,” five studies using the “Endoscopic specimen bag,” and four studies utilizing the “Specimen containment bag (Alexis Contained Extraction System, Applied Medical, Rancho Santa Margarita, CA, USA).” Other bag types employed across the studies included Ecosac, Lahey Bag, Anchor tissue retrieval system, and bowel bag (3M Steri-DrapeTM Isolation Bag 1003, St. Paul, MN, USA), 10- or 15 mm polyurethane specimen pouches (Covidien Surgical, Mansfield, MA, USA), and 32 × 41 cm nylon bags (4 L Espiner EcoSac morcellation, Espiner Medical) were deployed intra-abdominally. For larger uterine specimens, bigger bags without a frame (LapSac; Cook Medical, Bloomington, IN, USA) were used, although the placement and wrapping were more difficult and took much more time. In addition, some studies used a combination of a bag, Alexis’s ring, and stapler, whereas others did not specify the bag type used. These bag types were chosen based on the specific requirements of the procedures and preferences of the surgeons.

### Surgical procedures

The primary surgical procedures performed in these studies included a wide range of indications, with some of the most common being abnormal uterine bleeding, pelvic pain, dysmenorrhea, and dyspareunia. These studies also addressed various histologic types, including leiomyomas (uterine fibroids) and malignancies such as endometrioid adenocarcinoma and carcinosarcoma. Among these, leiomyomas were the most commonly addressed histologic types, and some cases also involved adenomyosis. Furthermore, the studies explored other malignancies like serous adenocarcinoma, carcinosarcoma, and clear-cell adenocarcinoma. These studies demonstrated a varied spectrum of procedures and indications, underscoring the versatility of transvaginal morcellation within enclosed bags.

### Duration of the procedure

The range of surgical procedure times varied widely, and the median surgical procedure time across these studies was approximately 120 min, with some studies reporting a range of 70–210 min. Notably, studies reported that the mean and median times for organ extraction and morcellation were approximately 13 and 16 min, respectively. A weak positive correlation was found between the duration of the procedure and the weight of the extraction tissue (Correlation Coefficient = 0.236). This suggests that longer procedure durations were associated with an increase in the weight of the extracted tissue. The overall trend suggests a reasonable duration for transvaginal morcellation within an enclosed bag, contingent on individual patient factors and surgeon experience. However, it is essential to consider individual patient factors, the complexity of the case, and the surgeon’s experience when assessing the suitability of this technique in practice.

### Blood loss

Out of the 22 studies analyzed, 12 provided data on blood loss. These data were collected from various surgical procedures, such as total laparoscopic hysterectomy, subtotal hysterectomy, robotic-assisted hysterectomy, and laparoscopic myomectomy, with the majority involving laparoscopic surgeries. In the Ding 2022 [16] study, it was observed that patients undergoing minimally invasive surgery with vaginal in-bag morcellation had significantly lower estimated blood loss compared to patients undergoing abdominal hysterectomy (*P* < 0.001). In the Meurs 2017 [25] study, which observed surgical outcomes across different morcellation methods (Power Morcellation, Vaginal Morcellation, and Mini-laparotomy), no significant difference in estimated blood loss was found among power morcellation, manual vaginal morcellation, and mini-laparotomy in 297 patients. In this study, containment bags were utilized in the majority of patients across all techniques. Multivariate regression analysis revealed no significant differences in blood loss, length of stay, or complications among the techniques. blood loss is not a major concern for using morcellation bags when compared between morcellation techniques.

### Malignancy risk

Among the 22 studies reviewed, 8 of them examined cases involving malignancy, while 3 of these studies were unsuspected malignancy. In the study conducted by Ding, which included a substantial cohort of 5683 low-risk benign patients, the incidence of malignancy was found to be remarkably low at just 0.33% [16]. Importantly, among the patients diagnosed with endometrial cancer in this study, three underwent in-bag manual morcellation, and none experienced relapse during a follow-up period spanning 32 months, ranging from 16 to 70 months. Similarly, in the Serur study [28]; which comprised 104 patients, 2 cases (1.92%) of occult malignancy were detected. Encouragingly, both individuals showed no signs of disease during follow-up periods of 28 and 36 months, respectively. Furthermore, in a separate study that involved unsuspected malignancy treated with power morcellation, no dissemination was observed. Even at the time of our current analysis, which is ≥ 2 years after the initial surgery, the patient remains free of recurrence.

### Weight of the extracted tissue

The range of extraction tissue weights varied widely from as low as 70 g to as high as 950 g. The median values suggest that the typical weight of the extracted tissue was approximately 210 g [13] and 372 g [17]. However, it is important to note that there were substantial variations in the reported values. In Cohen 2019 [15], a standard deviation of 465 ± 208 was provided, indicating a considerable degree of variability in the weights of the extracted tissue within that specific study.

### Reported adverse events

Ten studies have reported various adverse events. We have classified these adverse events based on their relatedness to the morcellation process (Table 3). Notably, among these ten studies, only three studies reported morcellation-related intraoperative complications. In Carrubba’s study conducted in 2020, it was observed that 5 patients (14.71%) required conversion to the abdominal route, highlighting the need for flexibility in surgical approaches. In addition, Serur’s 2016 study reported a conversion rate to an open surgical approach of 5.9% (7 out of 117 patients). Within this subgroup, one patient experienced a substantial estimated blood loss of 1200 mL during the procedure, which was attributed to bleeding from a broad ligament myoma. However, it is noteworthy that Serur’s study clarified that these complications were not directly attributable to the morcellation technique itself. In the study by Gil-Gimeno et al. in 2020 [22], complications were noted in two women. One case involved a posterior vaginal laceration during vaginal morcellation, while the other case included a bladder injury in a woman with a uterus weighing 1321 g. The latter incident was likely to have occurred during the initial bladder dissection rather than directly related to the morcellation procedure.

**Table 3:** Adverse events reported in the included studies

Study	Year	Number of patients	Complication type	Details
*Related to morcellation*				
Carrubba et al. [12]	2020	34	Conversion to abdominal route	Five patients (14.71%) required conversion
Serur et al. [28]	2016	117	Conversion to open surgery	Seven patients (5.9%) required conversion; 1 patient had substantial blood loss (1200 mL) due to bleeding from a broad ligament myoma
Gil-Gimeno et al. [22]	2020	79	Vaginal laceration, bladder injury	One case of posterior vaginal laceration during vaginal morcellation; 1 case of bladder injury during initial bladder dissection in a woman with a uterus weighing 1321 g
*Not related to morcellation*				
Cohen et al. [15]	2019	32	Various complications	One intraoperative serosal tear of the distal ileum (repaired without conversion); postoperative complications including pelvic abscess, low-grade complications, and incisional issues
Casarin et al. [13]	2023	10	Vaginal bleeding	One patient experienced postoperative vaginal bleeding and sought emergency care, but no pathologic findings were identified
Dotson et al. [17]	2018	88	Various complications	Thirty-day postoperative complications in seven patients, including vaginal cuff separation, port-site hematoma, port-site cellulitis, vaginal cuff cellulitis, bladder infection, pulmonary edema, and musculoskeletal injury
Favero et al. [19]	2012	8	Postoperative vesicovaginal fistula	One patient presented about 14 days after the procedure with a vesicovaginal fistula that required surgical repair
Favero et al. [20]	2015	30	Significant postoperative complications	Two patients (6%) experienced vesicovaginal fistula and vaginal cuff suture dehiscence, both requiring surgical correction
Meurs et al. [25]	2017	297	Various complications	Intraoperative complications included genitourinary tract injury, anesthesia-related event, and bleeding; two cases of vaginal laceration or cuff separation in vaginal morcellation group; two cases of small bowel obstruction and two cases of severe intra-abdominal infection in minilaparotomy group; low-grade urinary tract or wound infections; 1 patient with unsuspected grade 1 endometrial adenocarcinoma in PM group
Wyman et al. [31]	2012	100	Secondary surgery	One patient required postoperative secondary surgery

Meanwhile, the remaining non-morcellation-related intraoperative or postoperative adverse events were reported in different seven studies (Table 3). Cohen et al. (2019) detailed intraoperative and postoperative issues, including pelvic abscess and skin-related problems. Dotson et al. (2018) found multiple postoperative complications, such as vaginal cuff separation and bladder infections. Favero et al. (2012, 2015) documented vesicovaginal fistulas requiring surgical repair. Meurs et al. (2017) reported diverse complications, including genitourinary tract injuries and infections. In addition, Wyman et al. (2012) noted one patient requiring secondary surgery postoperatively.

## Discussion

The results from the comprehensive analysis of studies are reassuring about the usefulness of transvaginal morcellation within an enclosed bag in gynecological surgeries. The culmination of our research effort, comprising 22 carefully selected articles, has yielded valuable insights into the use of this technique. In the reviewed studies, various aspects were assessed, including the age and sample size of the participants, the type of bag used, histologic tissue type, surgical procedures, primary and secondary study outcomes, procedure duration, blood loss, and reported adverse effects. The primary focus of the analysis was to evaluate the safety and efficacy of transvaginal morcellation within an enclosed bag during gynecological surgeries. Furthermore, an examination was conducted to explore the potential for tumor recurrence and dissemination.

The reported blood loss varied, with minimum reported mean values ranging from 20 to 260 mL, as noted in the included studies. The median blood loss across these studies was approximately 110 mL, although some studies reported a range of 20–1200 mL. These findings suggest that blood loss can vary significantly among patients undergoing transvaginal morcellation within an enclosed bag. It is important to differentiate between blood loss occurring during the procedure and blood loss specifically due to the morcellation process. Factors such as the type of surgery, the size and condition of the uterus or fibroids, and the surgical technique employed may contribute to these variations. It is crucial for future studies to specifically assess blood loss attributable to morcellation and distinguish it from the overall blood loss that may be associated with other procedures of the operation.

Unfortunately, not all uterine cancers can be diagnosed preoperatively. Preoperative assessment of patient uterine masses or abnormal uterine bleeding must account for the limitations of endometrial biopsy and imaging studies in evaluating the possibility of uterine malignancy. Even in cases where benign indications are present, it is imperative to remain vigilant regarding the potential for unsuspected malignancy. In line with these facts, a cautious approach is recommended, particularly in instances involving bulky tumors, wherein power morcellation should be avoided in favor of manual morcellation [33]. Preoperative approaches to minimize the risk of inadvertently morcellating a malignant tumor include endometrial sampling, pelvic sonography, and MRI. Endometrial sampling detects endometrial carcinoma, while sonography is useful for the initial detection of fibroids but is limited in distinguishing benign from malignant masses [34, 35]. MRI offers detailed imaging of uterine masses, assessing tumor size, margins, and invasive growth, although it may struggle to differentiate leiomyosarcoma from benign leiomyoma [36]. Combining patients’ demographic information, medical history, and imaging techniques with serum tests and using advanced MRI sequences can enhance diagnostic accuracy and reduce the risk of morcellating malignant tumors [33].

While most of the included studies involving malignant indications or cases of unexpected malignancy, with a mean follow-up of 11.9 months, did not report tissue dissemination, it is worth noting that Dotson’s study did identify tumor spillage in 2 out of 52 cases (3.85%) involving malignancy [17]. In this context, the FDA recommends that patients be informed about the risk of occult cancer and the potential for laparoscopic power morcellators to disseminate cancer.

While the risk of dissemination appears low in most scenarios, meticulous attention to the choice of technique remains crucial to ensure patient safety and optimal outcomes. However, enclosed bag morcellation has potential advantages in terms of preventing the abdominal spread of tumor cells, compared to open morcellation, which can make it a useful option. Further studies should be conducted to assess the potential malignancy rate and the risk of tissue dissemination in cases of unsuspected malignancy.

Meanwhile, most studies indicated that no ruptures were observed in the bag. Only three articles reported bag ruptures [12, 15, 29]. Solima’s findings showed that approximately 33.33% (4 out of 12) of the EndoCatch bags had minimal ruptures. Cohen’s study examined the risk of spillage associated with different types of bags, and it found that the Endo Catch bag had the highest risk, with in vitro spillage observed in 32% (8 out of 25) bags. While Cohen Indicated that the bag’s leakage had no clinically significant impact. However, it is important to interpret these findings cautiously. Cohen et al. mentioned in their conclusion that they did not specifically assess the protective qualities of different specimen containment bags, nor did they account for potential variations in extraction methods. Therefore, it remains uncertain whether the observed leakage was solely attributable to the bag itself, the extraction technique used, or other unidentified factors. In light of these uncertainties, it should be noted that the studies reviewed did not explicitly investigate the bag’s integrity. Considering anatomic factors, it is reasonable to expect that vaginal morcellation could potentially lead to an increased risk of bag perforations.

While there may be variations in analgesia requirements between different morcellation methods included in the review, postoperative pain levels, as indicated by Visual Analog Scale (VAS) scores, generally remain comparable, suggesting that transvaginal morcellation does not lead to undue discomfort. The preservation of female sexual function is an important aspect, with one group showing no significant differences in postoperative sexual function scores, while another group did report some worsening. Furthermore, it is worth noting that associations between factors like uterine size, BMI, and the route of morcellation did not reveal any significant concerns in terms of safety. Notably, some studies have reported no complications or intraoperative issues related to transvaginal morcellation within an enclosed bag. It is important to note that the occurrence of adverse events varied among the studies, and the majority of patients did not experience complications.

This observation suggests that the overall safety profile of transvaginal morcellation within an enclosed bag is favorable when performed by experienced surgical teams. Nevertheless, it is important to consider the potential link between tumor weight and the occurrence of complications. Overall, the data highlight the variability in the weight of extraction tissue in gynecological surgeries. This variability could be influenced by factors such as the type of surgery, patient characteristics, the presence of pathology (e.g., fibroids or tumors), and the surgical technique employed (e.g., morcellation method). It is important for surgeons to consider these factors when planning and performing gynecological procedures involving tissue extraction to ensure the safety and effectiveness of the surgery. In addition, further analysis and comparisons, as indicated by the data point comparing abdominal and vaginal morcellation, may provide valuable insights into the factors affecting tissue weight and the choice of surgical approach.

### Strengths and limitations

This is the first systematic review evaluating the outcomes of contained transvaginal morcellation in gynecological surgeries. Five different databases were used in the search and a large number of studies were reviewed for inclusion. Unfortunately, access to the EMBASE database was not obtained during the execution of our study, and as a result, it was not incorporated into our search strategy for identifying relevant studies. We included all available observational and experimental studies with variations in sample sizes, so our results combine case report data with larger studies. The quality of the included studies was assessed rigorously and studies’ overall quality ranged from fair to good. In addition, PRISMA 2020 standards were followed to ensure a consistent methodology.

The FDA encourages peer-reviewed scientific literature and prompt reporting of adverse events related to contained morcellation including the spread of unexpected cancer. All this information was assessed in our review, which may help in the improvement of contained morcellation procedures and the development of new techniques with lower risks.

A notable limitation in our study was the considerable heterogeneity observed in certain outcomes, which proved challenging to mitigate. This variability may, in part, be attributed to differences in surgical methods employed, which can vary not only between individual surgeons but also across various surgical cases. Another limitation is the lack of detailed reporting on whether the uterus was left in situ or removed in the included studies, which prevented a more granular analysis of outcomes based on this criterion. This highlights the need for more comprehensive data reporting in future research to better compare different surgical modalities. This inherent heterogeneity underscores the need for more standardized trials in the field of gynecological surgeries involving in-bag morcellation. Enhanced standardization would not only raise the quality of data but also reduce heterogeneity, enabling a more robust and well-founded analysis. A concerted effort to establish standardized protocols and guidelines for conducting such trials worldwide is imperative. By doing so, we can elevate the level of confidence in the safety and efficacy of in-bag morcellation, facilitating informed and evidence-based decisions in clinical practice.

## Conclusion

The results of the collective analysis of various studies shed light on the safety and effectiveness of transvaginal morcellation within an enclosed bag in gynecological surgeries. Overall, the technique appears to offer several advantages, including reduced invasiveness, shorter operative times, and minimal blood loss, compared to traditional approaches such as abdominal hysterectomy. However, there are still concerns about the safety of the technique in cases of suspected or confirmed malignancy. In this regard, the risk of tumor recurrence or dissemination should also be assessed in conjunction with these findings and any available long-term follow-up data.

## Data Availability

Data is available upon reasonable request to the primary author.

## Declarations

### Registration on PROSPERO

https://www.crd.york.ac.uk/prospero/display_record.php?ID=CRD42023437418.

## References

[1] Mohiuddin K, Swanson SJ (2013) Maximizing the benefit of minimally invasive surgery. J Surg Oncol 108(5):315–319. 10.1002/jso.23398 24037974 10.1002/jso.23398

[2] Kho KA, Nezhat CH (2014) Evaluating the risks of electric uterine morcellation. JAMA 311(9):905. 10.1001/jama.2014.1093 24504415 10.1001/jama.2014.1093

[3] Park J-Y, Park S-K, Kim D, Kim J-H, Kim Y, Kim Y, Nam J (2011) The impact of tumor morcellation during surgery on the prognosis of patients with apparently early uterine leiomyosarcoma. Gynecol Oncol 122(2):255–259. 10.1016/j.ygyno.2011.04.021 21565389 10.1016/j.ygyno.2011.04.021

[4] Center for Devices and Radiological Health (2020) UPDATE: Perform only contained morcellation when laparoscopic power morcellation is appropriate: FDA Safety Communication. In: U.S. Food And Drug Administration. https://www.fda.gov/medical-devices/safety-communications/update-perform-only-contained-morcellation-when-laparoscopic-power-morcellation-appropriate-fda

[5] Taylan E, Şahin Ç, Zeybek B, Akdemir A (2017) Contained morcellation: review of current methods and future directions. Front Surg. 10.3389/fsurg.2017.00015 28352629 10.3389/fsurg.2017.00015 PMC5348539

[6] Abouzid A, Shetiwy M, Hassan A, Elghaffar MA (2022) Scalpel morcellation during laparoscopic hysterectomy for large uterine fibroids .Is it a safe alternative to power-morcellation? Indian J Surg 85(2):413–419. 10.1007/s12262-022-03424-1 10.1007/s12262-022-03424-1

[7] Donat LC, Clark M, Tower AM, Menderes G, Parkash V, Silasi D, Azodi M (2015) Transvaginal morcellation. JSLS 19(2):e2014.00255. 10.4293/jsls.2014.00255 10.4293/jsls.2014.00255 PMC4432719 26005318

[8] Page MJ, McKenzie JE, Bossuyt PM, Boutron I, Hoffmann T, Mulrow CD, Shamseer L, Tetzlaff J, Akl EA, Brennan S, Chou R, Glanville J, Grimshaw J, Hróbjartsson A, Lalu MM, Li T, Loder E, Mayo-Wilson E, McDonald S, McGuinness LA, Stewart L, Thomas J, Tricco AC, Welch V, Whiting P, Moher D (2021) The PRISMA 2020 statement: an updated guideline for reporting systematic reviews. Syst Rev. 10.1186/s13643-021-01626-4 33781348 10.1186/s13643-021-01626-4 PMC8008539

[9] Study Quality Assessment Tools | NHLBI, NIH. In: NHLBI, NIH. https://www.nhlbi.nih.gov/health-topics/study-quality-assessment-tools

[10] Higgins JPT, Altman DG, Gøtzsche PC, Jüni P, Moher D, Oxman AD, Savović J, Schulz KF, Weeks L, Sterne JaC (2011) The cochrane collaboration’s tool for assessing risk of bias in randomised trials. BMJ 343:d5928. 10.1136/bmj.d5928 22008217 10.1136/bmj.d5928 PMC3196245

[11] Akdemir A, Arı SA, Taylan E, Ökmen F, Şahin Ç (2022) Comparison of laparoscopic enclosed electromechanical morcellation and vaginal enclosed scalpel morcellation at laparoscopic myomectomy: a prospective randomized trial. J Obstet Gynaecol Res 49(2):691–700. 10.1111/jog.15507 36420685 10.1111/jog.15507

[12] Carrubba AR, Jijón A, Heckman MG, Brushaber D, Chen AH, Dinh TA, DeStephano CC (2020) Association of uterine dimensions and route of contained morcellation following laparoscopic hysterectomy. Minerva Ginecol. 10.23736/s0026-4784.20.04602-x 32677775 10.23736/s0026-4784.20.04602-x

[13] Casarin J, Ghezzi F, Dri M, Granato V, Laganà AS, Ambrosoli AL, Cromi A (2023) Laparoscopic subtotal hysterectomy followed by in-bag transvaginal corpus uteri morcellation and extraction: a case series. Eur J Obstet Gynecol Reprod Biol 282:124–127. 10.1016/j.ejogrb.2023.01.015 36708659 10.1016/j.ejogrb.2023.01.015

[14] Chandler J, Herman R, Winstead J, Mulla ZD (2016) It’s in the bag: laparoscopic approach to excision of large uterine specimens. J Minim Invasive Gynecol 23(7):S43. 10.1016/j.jmig.2016.08.109 10.1016/j.jmig.2016.08.109

[15] Cohen SL, Clark NV, Ajao MO, Brown D, Gargiulo AR, Gu X, Einarsson JI (2019) Prospective evaluation of manual morcellation techniques: minilaparotomy versus vaginal approach. J Minim Invasive Gynecol 26(4):702–708. 10.1016/j.jmig.2018.07.020 30075302 10.1016/j.jmig.2018.07.020

[16] Ding Y, Han Y, Zhang S, Shi X (2022) The incidence of unexpected uterine malignancies in hysterectomies carried out for benign indications. J Cancer Res Clin Oncol 149(8):4339–4345. 10.1007/s00432-022-04343-0 36083311 10.1007/s00432-022-04343-0 PMC10349766

[17] Dotson S, Landa A, Ehrisman J, Secord AA (2018) Safety and feasibility of contained uterine morcellation in women undergoing laparoscopic hysterectomy. Gynecol Oncol Res Pract. 10.1186/s40661-018-0065-1 30410774 10.1186/s40661-018-0065-1 PMC6208173

[18] English DP, Menderes G, Azodi M (2015) Controlled removal of a large uterus within a bowel bag and morcellation in the bowel bag from the vagina. Gynecol Oncol 137(3):589–590. 10.1016/j.ygyno.2015.03.014 25797081 10.1016/j.ygyno.2015.03.014

[19] Favero G, Antón C, Silva ASE, Ribeiro A, Araújo M, Miglino G, Baracat EC, Carvalho JP (2012) Vaginal morcellation: a new strategy for large gynecological malignant tumor extraction. Gynecol Oncol 126(3):443–447. 10.1016/j.ygyno.2012.05.023 22634019 10.1016/j.ygyno.2012.05.023

[20] Favero G, Miglino G, Köhler C, Pfiffer T, Silva ASE, Ribeiro A, Xin L, Antón C, Baracat EC, Carvalho JP (2015) Vaginal morcellation inside protective pouch: a safe strategy for uterine extration in cases of bulky endometrial cancers: operative and oncological safety of the method. J Minim Invasive Gynecol 22(6):938–943. 10.1016/j.jmig.2015.04.015 25917277 10.1016/j.jmig.2015.04.015

[21] Ghezzi F, Casarin J, Greco F, Puggina P, Uccella S, Serati M, Cromi A (2017) Transvaginal contained tissue extraction after laparoscopic myomectomy: a cohort study. BJOG Int J Obstet Gynaecol. 10.1111/1471-0528.14720 10.1111/1471-0528.14720 28467660

[22] Gil-Gimeno A, Laberge PY, Lemyre M, Gorak E, Maheux-Lacroix S (2020) Morcellation during total laparoscopic hysterectomies: implications of the use of a contained bag system. J Obstet Gynaecol Can 42(7):839–845. 10.1016/j.jogc.2019.11.004 32273084 10.1016/j.jogc.2019.11.004

[23] Günthert AR, Christmann-Schmid C, Kostov P, Mueller C (2015) Safe vaginal uterine morcellation following total laparoscopic hysterectomy. Am J Obstet Gynecol 212(4):546.e1-546.e4. 10.1016/j.ajog.2014.11.020 25460836 10.1016/j.ajog.2014.11.020

[24] Kliethermes CJ, Walsh TM, Guan Z, Guan X (2017) Vaginal tissue extraction made easy. J Minim Invasive Gynecol 24(5):726. 10.1016/j.jmig.2017.02.005 28232039 10.1016/j.jmig.2017.02.005

[25] Meurs EAIM, Brito LGO, Ajao MO, Goggins ER, Vitonis AF, Einarsson JI, Cohen SL (2017) Comparison of morcellation techniques at the time of laparoscopic hysterectomy and myomectomy. J Minim Invasive Gynecol 24(5):843–849. 10.1016/j.jmig.2017.04.023 28483536 10.1016/j.jmig.2017.04.023

[26] Montella F, Riboni F, Cosma S, Dealberti D, Prigione S, Pisani C, Rovetta E (2014) A safe method of vaginal longitudinal morcellation of bulky uterus with endometrial cancer in a bag at laparoscopy. Surg Endosc Other Interv Tech 28(6):1949–1953. 10.1007/s00464-014-3422-0 10.1007/s00464-014-3422-0 24566741

[27] Nakabayashi A, Odaira K, Horibe Y, Kanno T, Akizawa Y, Tabata T (2020) A case of unsuspected low-grade endometrial stromal sarcoma successfully treated with two minimally invasive surgeries. Gynecol Minim Invasive Ther 9(4):237. 10.4103/gmit.gmit_67_19 33312870 10.4103/gmit.gmit_67_19 PMC7713657

[28] Serur E, Zambrano N, Brown K, Clemetson E, Lakhi N (2016) Extracorporeal manual morcellation of very large uteri within an enclosed endoscopic bag: our 5-year experience. J Minim Invasive Gynecol 23(6):903–908. 10.1016/j.jmig.2016.03.016 27058770 10.1016/j.jmig.2016.03.016

[29] Solima E, Scagnelli G, Austoni V, Natale A, Bertulessi C, Busacca M, Vignali M (2015) Vaginal uterine morcellation within a specimen containment system: a study of bag Integrity. J Minim Invasive Gynecol 22(7):1244–1246. 10.1016/j.jmig.2015.07.007 26205578 10.1016/j.jmig.2015.07.007

[30] Takeda A, Koike W, Watanabe K (2018) Multimodal imaging for diagnosis and management of parasitic peritoneal myoma with myxoid degeneration after laparoscopic-assisted myomectomy with electric power morcellation. J Obstet Gynaecol Res 44(6):1163–1168. 10.1111/jog.13621 29516586 10.1111/jog.13621

[31] Wyman AM, Fuhrig L, Bedaiwy MA, Debernardo R, Coffey G (2012) A novel technique for transvaginal retrieval of enlarged pelvic viscera during minimally invasive surgery. Minim Invasive Surg 2012:1–4. 10.1155/2012/454120 10.1155/2012/454120 PMC3395334 22811899

[32] Zhang SJ, Guo L, Deng PZ, Dai SR, Ren Q, Tao X, Zhu W (2022) Application of transvaginal morcellation within disposable extraction bag with traction wire in laparoscopic myomectomy. Zhonghua Yi Xue Za Zhi 102(26):2030–2032. 10.3760/cma.j.cn112137-20220329-00655 35817729 10.3760/cma.j.cn112137-20220329-00655

[33] Hall T, Lee SI, Boruta DM, Goodman A (2015) Medical device safety and surgical dissemination of unrecognized uterine malignancy: morcellation in minimally invasive gynecologic surgery. Oncologist 20(11):1274–1282. 10.1634/theoncologist.2015-0061 26382742 10.1634/theoncologist.2015-0061 PMC4718440

[34] Bansal N, Herzog TJ, Burke W, Cohen CJ, Wright JD (2008) The utility of preoperative endometrial sampling for the detection of uterine sarcomas. Gynecol Oncol 110(1):43–48. 10.1016/j.ygyno.2008.02.026 18445505 10.1016/j.ygyno.2008.02.026

[35] Exacoustos C, Manganaro L, Zupi E (2014) Imaging for the evaluation of endometriosis and adenomyosis. Best practice and research. Clin Obstet Gynaecol 28(5):655–681. 10.1016/j.bpobgyn.2014.04.010 10.1016/j.bpobgyn.2014.04.010 24861247

[36] Cornfeld D, Israel G, Martel M, Weinreb J, Schwartz P, McCarthy S (2010) MRI appearance of mesenchymal tumors of the uterus. Eur J Radiol 74(1):241–249. 10.1016/j.ejrad.2009.03.005 19349135 10.1016/j.ejrad.2009.03.005

